# Ethical learning on international medical electives: a case-based analysis of medical student learning experiences

**DOI:** 10.1186/s12909-018-1181-7

**Published:** 2018-04-11

**Authors:** Gemma Bowsher, Laura Parry-Billings, Anna Georgeson, Paula Baraitser

**Affiliations:** King’s Centre for Global Health, Suite 2.13 Weston Education Centre, Cutcombe Road, London, SE5 9RJ UK

**Keywords:** medical elective, ethical learning, transformative learning, Mezirow, lobal health education

## Abstract

**Background:**

Students on international medical electives face complex ethical issues when undertaking clinical work. The variety of elective destinations and the culturally specific nature of clinical ethical issues suggest that pre-elective preparation could be supplemented by in-elective support.

**Methods:**

An online, asynchronous, case-based discussion was piloted to support ethical learning on medical student electives. We developed six scenarios from elective diaries to stimulate peer-facilitated discussions during electives. We evaluated the transcripts to assess whether transformative, experiential learning took place, assessing specifically for indications that 1) critical reflection, 2) reflective action and 3) reflective learning were taking place. We also completed a qualitative thematic content analysis of the discussions.

**Results:**

Of forty-one extended comments, nine responses showed evidence of transformative learning (Mezirow stage three). The thematic analysis identified five themes: adopting a position on ethical issues without overt analysis; presenting issues in terms of their effects on students’ ability to complete tasks; describing local contexts and colleagues as “other”; difficulty navigating between individual and structural issues, and overestimation of the impact of individual action on structures and processes.

**Conclusion:**

Results suggest a need to: frame ethical learning on elective so that it builds on earlier ethical programmes in the curriculum, and encourages students to adopt structured approaches to complex ethical issues including cross-cultural negotiation and to enhance global health training within the curriculum.

## Background

Electives are student-organised placements within the medical curriculum [1]. Ninety per cent of UK medical students go abroad for their mandatory elective [2] and four fifths of these students choose to spend time in a low- or middle-income country [3].

Electives offer experiential learning through exposure to different models of heath care, unfamiliar professional cultures and new social contexts [4]. They may encourage transformative learning [5] whereby students become more aware of the assumptions they make regarding unfamiliar situations, and question and modify these in response [6, 7].

One way in which electives may stimulate both experiential and transformative learning is by presenting ethically complex situations, such as opportunities to practice beyond competence [8, 9], exposure to alternative ethical paradigms and use of scarce local resources for learning [10]. Rahim et al. (2016) have described 13 ethical situations that medical students should be prepared to manage on elective [11].

Ethical training before the elective is considered mandatory; however, learning strategies are diverse with little evaluation of learning outcomes [5, 11, 12]. Although learning theory suggests that feedback to support reflection during the elective will facilitate learning [13, 14], little has been published on educational support for students on placements [11].

This study investigates the impact of a structured programme delivered online to support learning on ethical issues during an eight-week elective placement. Using a learning support programme delivered via asynchronous, online, peer-facilitated discussion of ethically complex case studies. This study examines whether such a programme can assist students on their electives undergo Mezirow’s Transformative learning, and develop skills in 1) critical reflection, 2) reflective action and 3) reflective learning [6, 7]. Case-based learning is recommended as a strategy to support ethical learning on electives [15, 8, 10, 16–20].

The transcripts from the peer-facilitated discussions were studied in order to look for evidence of transformative learning and to conduct a thematic analysis.

Ethical Approval was granted by the King’s College London Research Ethics Board. Consent from participants was gained orally since recruitment took place via distance methods such as skype. This was approved by the ethics board.

## Methods

We developed the online intervention based on: a review of the literature on learning interventions to support ethical practice on electives [11]; interviews with students before, during and after electives and elective learning diaries completed by students on electives. From this first stage of work, we identified and developed six ethical case studies to illustrate common and important issues experienced on electives. We collected these examples from previous research eliciting challenges experienced by students from King’s College London on their medical electives [11]. It was thought that by providing outgoing students with concrete examples of previous and likely ethical dilemmas from their medical school colleagues, they would be encouraged to examine their behaviours and assumptions, and use these examples as a stimulus to consider their own options for reflective action. Additionally, two ethical frameworks were selected from the published literature to support the management of ethical issues in unfamiliar contexts and to guide the peer-led discussion in a structured manner. One of these, the traditional ethical framework taught in medical schools, structures the evaluation of ethical issues using the themes of autonomy, non-maleficence, beneficence and justice [21]. This framework was selected because of its scope of application in clinical contexts, as well as its existing familiarity to the medical student participants who would have encountered these principles throughout their medical school curriculum at King’s College London (KCL) and who would have been expected to have internalised its meanings into daily practice. The second framework, developed specifically for electives, structures the discussion of ethical issues under the headings; humility, introspection, social justice and solidarity [22]. The addition of this framework served to structure discussion in particular relation to the role of the student practicing in settings where clinical medicine interacts with political legacies, power imbalances and inequitable health and social outcomes. It was felt that this would extend their learning by offering new and unfamiliar material to examine in the context of their experiences alongside the familiar traditional framework.

Participants (*n* = 19) in the programme were 4th year medical students from King’s College London. Recruitment was based on voluntary participation in the programme, but was offered to all, four hundred, departing elective students. Advertising took place via email and in-person at student meetings and events within the medical school for both facilitator and participant volunteers. The nineteen volunteers were all fourth year medical students about to depart on their elective, with variable experiences of previous international medical experiences. We recruited six peer facilitators who received four training sessions, with structured practice in group facilitation and ethics teaching. Two of the facilitators were also on their elective during the programme. The peer facilitators, although medical students each had more experience in global health than the average departing elective student; experience consisted of having undertaken special study modules in global health or a Bsc in global health. These facilitators were therefore more familiar with the application and critical evaluation of ethical scenarios in complex health settings. The peer facilitators managed the online case based discussions over the eight weeks with regular support from faculty, and were encouraged to facilitate discussion to build on the provided case studies in order to generate reflective and critical discussion of group experiences and ethical challenges. The discussion took place on a private area of a secure social networking platform. During the eight-week elective period (July–September, 2014) facilitators introduced a new ethical case for discussion at the beginning of each week with the ethical frameworks to support analysis. Frameworks were introduced at the earliest stage of the programme to allow students the duration of the intervention to further reflect and apply critical tools to the scenarios they were encountering. Facilitators introduced each week with the case and an open invitation to input ideas relating to the case-study and ethical frameworks, before gradually structuring conversation around a worked example either contributed by participants from their own experiences, or proposed by the facilitator. We chose asynchronous, online learning because it does not require participants to be present at the same time in the same location, a format that particularly suits the needs of elective students scattered widely across the globe. It enables students to self-direct their activity and to determine their level of involvement [23].

Nineteen participants were recruited to the study. Participants travelled all over the world including; Papua New Guinea, South Africa, Kenya, USA, India, Fiji, Zimbabwe and Malawi. Participants were encouraged to comment on the case study using the ethical frameworks, and to apply it to their own experiences. Our data set for this analysis was the complete transcripts of all the discussions that took place during the eight-week elective period July–September 2014 (Table 1).

**Table 1:** Case Studies for Weekly Online Discussion

Ethical Theme	Case Study
1. Uncertainty about how best to help	I am working at a TB clinic in West Africa. Yesterday the clinic ran out of gloves. I have brought some of my own with me, but not enough to share with the entire local staff working at the clinic. The local staff must continue to attend the clinic even without gloves. I am not sure what I should do in this situation?
2. Perceptions of Western medical students	A patient asked to see the ‘western doctor’ (me), rather than the local doctor. I explained that I was a medical student, not a doctor, but the patient still insisted on being examined by me.
3. Moving beyond one’s scope of practice	I am on an elective in South Africa. The registrar asked me to assist with an emergency C-section as there was no one else available to help. I have never done this type of procedure before, and remembered that the medical school advised us not to assist with a C-section as it is a high-risk exposure prone procedure.
4. Navigating different cultures of medicine	I am on elective in a busy rural hospital. On the labour ward, the midwives often shout at the patients, and even slap them on the face if they are making too much noise. Should I intervene?
5: One sided benefits	I am working in an outpatient gynaecological clinic. I am getting lots of practice doing examinations. I always ask for permission to do an examination, but my language skills are not very good. I think the patients may think that I am a doctor (not a medical student), which is why they let me examine them. I ask the nurse to come and explain in the local language and although she is very busy attending to patients, she obliges.

**Fig. 1:**
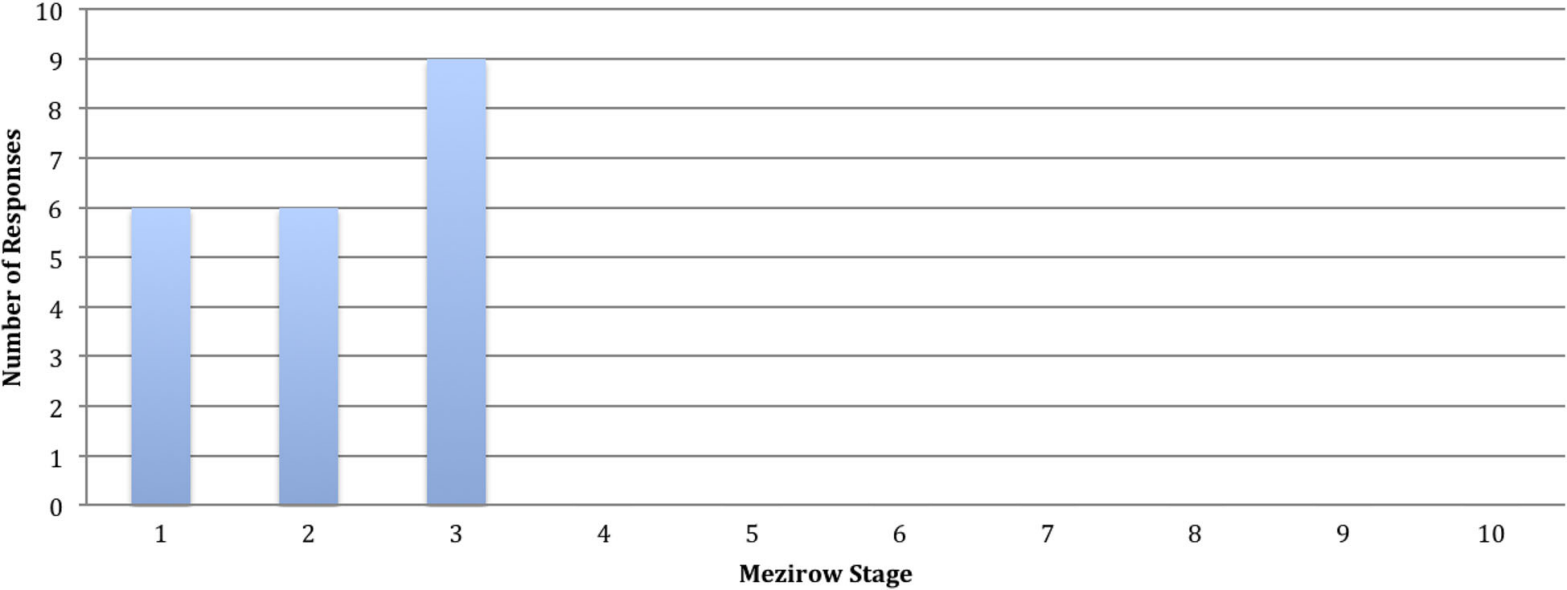
Distribution of coded data across the stages of Mezirow’s framework

The intervention uses Mezirow’s extensive work on transformative learning [6, 7] to establish the key learning objectives of 1) critical reflection, 2) reflective action and 3) reflective learning [24]. The common pathway for each of these learning objectives is that the intervention should challenge the assumptions present in existing meaning perspectives. Meaning perspectives are defined as ‘the structure of assumptions within which new experience is assimilated and transformed by one’s past experience during the process of interpretation...defined as higher-order schemata, theories, propositions, beliefs’. Mezirow asserts that a crucial element of adult learning is the process of contemplation that determines whether prior learning, based upon such meaning perspectives, is fit for purpose under present exigencies - this process providing a useful definition of ‘reflection’ for this intervention.

Furthermore it is feasible to consider the medical elective as an opportunity to examine reflection in the particular category of learning that Mezirow describes as communicative learning; “less a matter of testing hypotheses than of searching, often intuitively, for themes and metaphors by which to fit the unfamiliar into a meaning perspective, so that an interpretation in context becomes possible” [24]. Reflection in such circumstances requires the analysis of Meaning in terms of communicated ideals, values and ethics whilst contemplating pre-existing assumptions in order to evaluate the conditions within which such meanings continue to hold or necessitate challenge. Mezirow’s framework of Transformative learning is considered a procedural framework for conceptualising these goals of critical reflection, reflective action and finally reflective learning. Critical reflection can termed as ‘challenging the validity of presuppositions in prior learning’, which corresponds to Stages 3&4 of the transformative learning framework. Reflective action builds upon critical reflection by constituting action that follows the critical assessment of assumptions and corresponds to stages 5–8. Reflective learning refers to the total process of challenging assumptions and initiating novel action, however the emphasis is on the final stages of the Mezirow framework - Stages 9 & 10 - which terminate by a reordering of meaning perspectives integrated into one’s life on the basis of the catalysing context (Stage 1 & 2).

The intervention was designed to encourage students to undergo the process of transformative learning, with a particular focus on enabling reflection. Habermas has defined non-reflective learning as that which “takes place in action contexts in which implicitly raised theoretical and practical validity claims are naively taken for granted and accepted or rejected without discursive consideration” [25]. The online platform and intervention as a whole offers the antithesis to Habermas’ definition; 1) The weekly case study and ethical framework propositions transfer theoretical and practical validity claims from the implicit to the explicit realm 2) facilitators were trained specifically to challenge the assumptions and internalised norms of medical students encountering these ethical cases and their own ethical dilemmas 3) The process of facilitation and the online discussion space itself necessitate discursive consideration of “action contexts”.

### Analysis

The analysis of the online transcripts used both Mezirow’s framework of transformative learning and Kolb’s framework for experiential learning, to map the content of the online discussions to the suggested processes of learning [6, 26]. The first five stages (phase 1) correspond to the reflective aspects of Kolb’s learning pathway, whilst the latter five stages (phase 2) correspond to the action stages. All of the transcripts were coded line by line with reference to the stages listed in Table 2. The data were coded by two researchers independently and then cross-checked with any areas of disagreement resolved through discussion. In the third stage, the coded data was presented to a third researcher for final validation, and any remaining areas of uncertainty were discussed by all three researchers and finally resolved. The coded data excluded facilitator comments except for those provided by the two facilitators on their medical elective who were reflecting on their experiences simultaneously.

**Table 2:** Framework Stages Mezirow & Kolb

Stage	Mezirow’s Framework Stage	Kolb’s Experiential Learning Pathway Phase	Learning Phase
1	Disorienting Dilemma	Concrete, Reflective	Phase 1: Reflective
2	Self-Examination (Often with feeling of guilt or shame)		
3	Critical Assessment of epistemic, sociocultural or psychic assumptions	Abstract, Reflective	
4	Recognition of connection between one’s discontent and the process of transformation		
5	Explaining option for new behavior		
6	Planning a course of action	Abstract, Active	Phase 2: Active
7	Knowledge to implement plans		
8	Experimenting with new roles	Concrete, Active	
9	Building of confidence with new roles		
10	Reintegration into one’s life on the basis of conditions dictated by ones new life		

It was considered that Mezirow’s concept of meaning perspectives was a rich foundation for an examination of how students negotiate and construct their identities as learners facing complex ethical challenges in novel clinical settings. A thematic analysis was therefore undertaken to interrogate these dimensions and to explain students’ progress and obstacles through the learning process. The transcripts were read and re-read, focusing on content, style and narrative to derive themes from the online discussion in an open and reflexive manner [27]. This is an open and flexible form of qualitative analysis that removes categorical constraints whilst enabling interpretation of conscious thought and self-awareness along multiple theoretical lines [28].

## Results

### Mezirow

The participants made forty-one extended comments, each between 2 and 8 lines long to the online forum. The responses of two of the facilitators were included since they were also on their electives during the programme and contributed to the discussion more as participants than facilitators, sharing their experiences of their elective. Facilitator accounts accounted for three coded responses. The graph below presents the data coded according to the Mezirow framework – twenty-one posts constituted a clear domain within the Mezirow framework (Fig. 1).

While nine comments suggested that students critically assessed their assumptions, no material was found to suggest that this resulted in assumption modification. Table 3 demonstrates the progression through the first three stages of a participant, arresting at critical assessment. The student is responding to case study four, which has recalled to her a previous clinical experience abroad. She is uncomfortable about what she has observed and describes her attempts to mitigate the problem by providing an extra pair of hands but concludes that she is not in a position to comment on the situation. She makes no reference to the causes of, or possible solutions to the problem. Nor does she consider its impact on her own thinking or relation to her future practice.

**Table 3:** Example Stages of Progression in Mezirow Framework

Mezirow Stage	Participant Response
Disorienting Dilemma	Whilst working in an ex-orphanage in Romania I had to come to terms with the practice of local staff of restraining the residents with strait jackets and tying people to chairs. This situation had arisen due to a severe lack of staff and the complex physical and mental disabilities of the residents…
Self-Examination	It was extremely distressing to see and although we tried to prevent this happening as much as possible, by offering extra pairs of hands and distraction, there were often times when it seemed there was no alternative.
Critical Assessment	To change things long term is far easier said than done though and also calls into question whether or not we are qualified to make these sort of comments, whether we have any right to intervene in a workplace where we do not have to work day in day out.

### Emergent themes

#### Theme one: a tendency to adopt a position on ethical issues without overt engagement in a process of analysis

Participants had a tendency to adopt ethical positions and plan actions without connecting with the ethical frameworks provided and therefore without overt consideration of alternative responses.

*“Shouting to a certain extent can be accepted, but slapping is a physical abuse that we should not tolerate”.*

“*I think the medical student should wear his own gloves to attend the clinic. His own safety should be the highest priority for his elective. If there is an increased risk of transmission, due to the decreased infection control, I think it is sensible for the medical student to skip this clinic: For the local staff, if they absolutely have to continue the clinic, then straight hand hygiene has to be done.”*

These responses show a conversational style that offers personal views rather than a more structured response. It does not overtly acknowledge uncertainty or seek consultation and implies a lack of familiarity with standard processes for addressing a complex ethical situation in medical practice more generally. The ethical positions adopted were often framed as pragmatism within resource constrained situations to justify behaviour that might be unacceptable at home.

“*Why not assist?!...students assist with C-sections here because otherwise there would be no one else”.*

*“Whilst I have my own gloves I agree that the overall difference it [sharing the gloves] would make would be minimal. Whilst this may be a selfish thought, I feel that I need to keep the gloves for myself, as I don't know what situations I may encounter during the rest of the stay.”*

#### Theme two: a tendency to present issues in terms of their effects on students’ ability to complete medical tasks

Participants’ discussions of day-to-day issues in their elective settings focused primarily on the difficulties experienced completing their medical tasks. Issues of cultural competency were often characterised in terms of how they affected the student’s placement rather than the relevance of such issues to interpersonal dynamics in the clinical consultation.

*“Either way if such poor treatment were to continue (midwives shouting at patients) I would not be able to remain in that environment and would request to be moved to another department”.*

The issue of a language barrier was a commonly cited difficulty. Accounts suggested a one-sided discourse in the clinical setting, where language barriers were presented in terms of their impact on students’ ability to elicit a medical history and conduct an examination.

*“I've found that exaggerated actions and hand gestures tend to reinforce the rudimentary list of words that I slowly acquired while watching the other doctors go about their tasks. Armed with a Google Translate app on my phone, and with patients who understood the language barrier and hence spoke more slowly, this allowed for a tedious but still complete history taking, complete with trainee disclaimer at the start. Besides that, physical examination findings are hard signs and less likely to be affected by language barriers, thankfully.”*

No students raised the language barrier as a disorienting dilemma, despite the indication from the text above that practice in this regard diverges from what students are used to in the UK. There was no mention of the dynamics of language in the consultation, the complexity of issues relating to consent, or the influence of the language barrier on the patients’ experience of the consultation and caregiving.

#### Theme three: describing local contexts and colleagues as “other”

Participants frequently discussed issues affecting the “locals” using terminology with colonial and paternalistic implications to describe cultural practices, community groups, patients and health professionals.

*“I think that humility is very important in this situation…but something that I’ve found quite difficult to convey, or for the locals to understand.*”

“*If the hospital had better finances, I suspect there would be curtains for the patients. They are all aware of how it is in the west (some of the doctors were big fans of house) yet none of them seemed concerned by the lack of privacy, suggesting there is a strong cultural element.”*

These modes of speech, whilst unintentional, assume a hierarchy of knowledge and power between visiting students and local hosts. Participants switch between ethnocentric and culturally relativist arguments and actions without recognition or analysis of their assumptions.

#### Theme four: difficulty navigating between individual and structural issues, and an overestimation of the impact of individual action on structures and processes

Participants struggled to link the resource constrained clinical contexts that they observed in individual health institutions to the difficulties facing national health systems or international issues such as health care worker migration. They sometimes did not differentiate between challenges within individual institutions, countries or whole continents and there was a lack of critical analysis of the structural causes of the conditions faced and the students’ place within these.

*“The main problem here is the increased transmitting risk due to compromised infection control. And this is probably due to lack of resources in Africa”.*

Especially lacking was an appreciation of the tensions implicit in medical hegemony in diverse settings. Participants were sometimes unquestioning about medicine’s entanglements with colonial histories and cultural imposition, particularly in their view of medicine as an unquestionable, ahistorical, value-neutral good across post-colonial cultures and societies. Responses demonstrated the conflation of medical progress with modernity, and a sense that they had personal influence over systemic conditions due to their role as a visiting health professional.

*“This could be an opportunity to change one person’s attitude which in turn may spread through the community eg. explaining that some of the answers to their problems can be solved and helped at the local level rather than looking to the West to encourage sustainable and local development”.*

## Discussion

We did not find evidence that medical students on our ethical electives programme went through a process of transformative learning as defined by Mezirow, in response to the complex ethical issues they experienced on electives. While students reflected on ethical issues, we found limited evidence of questioning or modification of their views. Using Mezirow’s concept of meaning perspectives that consist of meaning structures (specific concepts, beliefs and judgements) and meaning perspectives (underlying assumptions about the way the world works) [27, 29], we found no evidence of a change in meaning perspectives. We also found tendency to plan without analysis even with tools to structure this process and opportunities for discussion to support reflection. We found little evidence of the construction of new knowledge or the application of what was learnt, which is an important part of experiential learning [26]. This finding is inconsistent with self-assessment by students of their professional and personal development during electives [30].

Our findings suggest three possible explanations. Our intervention may have been ineffective at supporting critical reflection and the use of ethical frameworks to structure thinking about ethical dilemmas; our students may have lacked familiarity with similar processes so that they were unable to benefit from the intervention, or that learning did take place but was not reflected in the discussions that we observed.

The materials used in our intervention were developed from ethical dilemmas reported by our students from previous elective placements and the pedagogical approach was informed by a review of the literature on similar interventions [11]. The intervention focused specifically on the analysis of ethical issues. Since reflection in experiential learning is supported by an understanding of the issues observed [13], learning on this programme may have benefited from more global health context to help students relate their experience to the global and structural causes of health inequality [31].

Our data suggests that students lacked familiarity with analysis of complex ethical situations in general and demonstrated limited engagement with the analytic frameworks provided. The frameworks were considered practical tools to aid recollection and discussion by students. The traditional framework [21] in particular was selected on the basis of its central integration into the KCL medical school curriculum, and as a recurrent theme in UK clinical practice. It was considered that this framework would constitute the basis of some assumptions for critical examination, as well as a familiar toolkit to initiate discussion approaching complex ethical scenarios. Students would previously have been expected to use this framework in medical school tutorials commencing in Year 1 to address ethical scenarios in the UK Health System involving dilemmas such as organ transplant, capacity decisions and end of life care. Two considerations emerge from the reluctance to engage with this framework, the first being a possible difficulty transferring the processes of reflection to alternative cultural contexts, and the second, which also accounts for the resistance to engaging with the second ethical framework [22], is described by Kleinman (2011) who has explored medical students’ struggle to reconcile “divided emotions” and “conflicted values” and the challenges within medical curricula to develop the skills to critique conflicts encountered in clinical settings [31]. He describes the students’ transition from a “pre-cynical” state in early medical training, to a “cynical” state on entering wards and clinics; a state that is numbed to personal subjectivity and neglects the moral boundaries of challenging moments in practice [32]. This theory is supported by work that has found medical students quick to adopt fairly fixed methods of learning, that are unresponsive to novel methods of learning introduced later in curricula [33]. Interventions designed to encourage the practice of reflection purely within the elective would ideally build on similar interventions delivered earlier in the medical curriculum, which is supported by other programmes integrating ethics training within the core curriculum [31, 34].

The online chat format of the intervention is likely to have encouraged an informal, conversational style that bypassed some of the more formal analysis that we aimed to stimulate. The intervention may have benefitted from more explicit reference to the range of action possible in response to complex ethical scenarios including questioning and modifying assumptions, witnessing and informal discussion with colleagues and peers. Striking the balance between adherence to the ethical frameworks and structured discussion that produces reflective responses may have been achieved with more skillful facilitation. The process for training facilitators may not have adequately addressed the complexity of this task and perhaps it might be fruitful to employ skilled professionals rather than peer-facilitators in this role given the various time and geographic complexities of the intervention.

The thematic analysis generated important insights and exposed some contradictory behaviour. For example theme two ‘*A Tendency to Present Issues in Terms of their Effects on Students’ ability to Complete Medical Tasks’* demonstrated some desire to avoid challenging ethical dilemmas “*if such poor treatment were to continue (midwives shouting at patients) I would not be able to remain in that environment and would request to be moved to another department”,* versus theme four which suggests that some students overestimate the impact of their individual action on structures and processes. It is possible that these contradictions emerge as students grapple with the daily ethical challenges found in clinical interactions, and the gulf between these and their perceptions of the root causes of systemic issues of ‘development’. Students tended to characterize behaviours and interpersonal engagements encountered in clinical scenarios as problems of culture, yet lacked the tools to interrogate why and how culturally relativist and ethnocentric arguments came into tension with their own meaning perspectives. Rather than engaging with such complex notions, the result was usually immediate action to remove themself from situations felt to compromise internalised ideas and assumptions. Interestingly broader issues conceptualised as those of ‘development’ and removed from individual behavior or “cultural practices” were felt to be inviting entry points for discussion and productive intervention. Clinical protocols enacted on the basis of resource shortages or limited technical capacity were seen as natural moments for students to offer their explanations of what they perceived to be the correct mode of practice on the basis of their familiarity with “western” – and implicitly “correct” - practices of biomedicine. Such behavior further displays the divorce between medical students’ appreciation of biomedicine as its own ‘cultural practice’ and cements a view of biomedicine as an entirely acultural endeavor which should best be evaluated by those with access the highest standards of medical modernity displayed in the global North.

The unfamiliarity of the elective situation may have both advantages and disadvantages for learning. Working in different contexts for short periods of time may highlight student assumptions about ethics and professionalism but it may also discourage engagement as students label the context and protagonists as ‘other’ and struggle to apply familiar ethical frameworks or link what they see to practice in more familiar environments. Whether this is an issue specific to elective situations is uncertain – certainly the medical curriculum provides limited opportunities to develop skills in the critical evaluation of political, social and historical dynamics as they interweave with present health concerns. Aptitude and engagement in this respect may currently be a function of individual interest rather than general skills developed during structured medical training. Our intervention did not provide opportunities for reflection or discussion in collaboration with host colleagues and peers that might have supported learning by adding a wider range of perspectives to the discussion.

There are a number of limitations to this study; the primary being the short eight week duration captured by this intervention, which assumes a detectable change occurring during or in the period after returning from elective once they have been reintroduced to their ‘home’ clinical setting. It may be that ethical learning continues over a longer duration and this may be captured by future studies that include an opportunity for reflection after the elective experience has terminated in a focus group setting. Small sample size is another limitation, with only 19 students volunteering for our intervention programme. A larger group may be able to demonstrate changes in ethical learning, more dynamic discussion or at least greater heterogeneity of experience. It would be important to understand medical student reluctance to participation in voluntary ethical learning programmes in order to expand the sample size. Expansion would minimise confounding factors by participant characteristics such as pre-intervention baseline, prior ethical training and relative levels of interest in ethical learning. The small number of programme volunteers may indicate a general lack of willingness amongst the medical student cohort to voluntarily engage with ethical teaching as described by Kleinman [32], and be indicative of systemic challenges within the medical school curriculum. The voluntary nature of the programme may have further contributed to the relatively limited conversations between participants; without a compulsory component of ethics engagement during the elective process, even some of those participants who volunteered contributed only cursory input.

The use of the ethical frameworks may have limited the breadth of student reflection, or even assumed a linear process of reflective development for the participants engaging with unfamiliar cases and experiences. The frameworks were proposed to ground discussion, but broadening the facilitation brief to engage with frameworks in a looser way whilst encouraging critical and reflective practice may generate more positive examples of transformative practice. The use of Mezirow’s framework, whilst not intended to rigidly encapsulate discussion content, may unfairly judge participants as failing to meet specific requirements in discussion – for this reason the thematic analysis serves as an illuminative adjunct to broaden the explanatory scope of the intervention and feed into future iterations of ethical learning programme on elective.

## Conclusion

Learning about the relationship between ethics and professionalism, global health and clinical practice is fundamental to making sense of the elective placement. The practices of critical reflection, reflective action and reflective learning are essential practices for understanding the unfamiliar and frequently challenging scenarios encountered as medical students move from UK medical contexts to settings abroad that exist with different cultures, pressures and practices. Learning from these encounters necessitates the interrogation of pre-existing assumptions as the fundamental starting point. The results from this project suggests that students struggle greatly with this process and consequently engage in a variety of problematic and contradictory practices which compound the need to further develop these skills in elective interventions and earlier in medical curricula. Future interventions need to frame ethical learning on electives so that they build on previous and more profound ethical training within the curriculum; making explicit links between complex ethical issues in familiar and unfamiliar contexts; supported by global health teaching that introduces the structural determinants of health, cultures of health and health care systems; and including host colleagues and peers in the conversation.

## Abbreviations

KCL: King’s College London
